# D_3_‐Creatine dilution for body composition assessment: A direct take on the matter

**DOI:** 10.1002/jcsm.13088

**Published:** 2022-09-26

**Authors:** Carla M. Prado, Stephan von Haehling

**Affiliations:** ^1^ Human Nutrition Research Unit, Department of Agricultural, Food and Nutritional Science University of Alberta Edmonton Alberta Canada; ^2^ Department of Cardiology and Pneumology University of Göttingen Medical Center Göttingen Germany; ^3^ German Centre for Cardiovascular Research (DZHK), Partner Site Göttingen Göttingen Germany

The prevalence and impact of low skeletal muscle mass (SMM) across healthy and disease states has positioned body composition as an impactful research field over the past decade, with an exponential number of publications in various specialty journals. As such, the quest to develop accurate, precise and practical techniques to assess SMM is of major importance, albeit a significant barrier to overcome.

The creatine‐(*methyl*‐d_3_) dilution, or D_3_‐creatine dilution (D_3_‐Cr) method, is an approach for SMM quantification of recent revitalization. It involves the consumption of deuterium‐labelled (stable) isotopes to estimate the creatine pool size, and hence SMM, assuming a constant value for whole‐body SMM creatine concentration.^1^, ^2^ A single fasting spot urine collection is needed 2‐ to 4‐days after dosing, which is analysed using high‐performance liquid chromatography. The growing interest in the use of D_3_‐Cr for body composition assessment is understandable. D_3_‐Cr is simple, safe, precise and of minimal subject burden including for neonates and bedridden/clinical populations; it is also practical for remote assessments.^1^, ^3^ Furthermore, SMM estimated from D_3_‐Cr is associated with physical performance, incidence of falls, fractures and mobility limitations in older adults.^4^, ^5^

Notwithstanding the important positive aspects of the D_3_‐Cr method, it is not without limitations, which were addressed in a comprehensive review by McCarthy et al.^1^ In this paper, the authors credit the decades of historical developments that led to the current use and understanding of the D_3_‐Cr method and consider its physiological premises. Further, they provide an intriguing and compelling discussion on methodological assumptions and therefore setbacks of the technique.^1^ These include considerations regarding the validity of the D_3_‐Cr method, and the actual body composition compartment measured, with both topics interpreted considering modern evidence from skeletal muscle research. In a snapshot, the authors caution against the use of D_3_‐Cr as a reference method for SMM assessment, highlighting potential sources of measurement error. As with other body composition techniques, these are mainly related to inherent assumptions used to estimate/quantify the compartment of interest, in this case, SMM. D_3_‐Cr estimates SMM from creatine pool size, which in turn is impacted by physiological premises of D_3_‐Cr absorption, distribution, catabolism and excretion.^1^ McCarthy et al.^1^ further highlight that SMM creatine concentration is not constant and varies between muscles and muscle composition, age, disease states and dietary patterns.

As two of their key takeaway messages, McCarthy et al.^1^ argue that D_3_‐Cr is not a direct measurement of SMM nor a direct measure of ‘functional’ muscle mass. Rather, D_3_‐Cr, as an indirect method, measures skeletal muscle contractile (myofibre) mass; it is therefore quantifying the muscle fibre component.^1^ Muscle function derives mainly from myofibres but also includes the actions of other intact muscle tissue components. The relatively large contribution of myofibres to muscle function may explain the good associations observed between D_3_‐Cr‐estimated SMM with functional/clinical outcomes, as shown in previously cited studies above.

The article by McCarthy et al.^1^ challenges body composition researchers to improve and further develop the use of D_3_‐Cr for SMM estimation and provide suggestions on how this can be achieved. We anxiously await for further advances on the use and applicability of this technique, including its relevance and practicality in clinical settings.^6^, ^7^, ^8^ Taking the article by McCarthy et al.^1^ into consideration, this journey may be long and end in frustration.

## Conflicts of interest

CMP reports three ongoing studies using/related to the D_3_‐Cr dilution method. Outside the submitted work, CMP reports receiving honoraria and/or paid consultancy from Abbott Nutrition, Danone, Nestle Health Science, Fresenius Kabi, Pfizer and AMRA Medical. SvH has been a paid consultant for and/or received honoraria payments from AstraZeneca, Bayer, Boehringer Ingelheim, BRAHMS, Chugai, Grünenthal, Helsinn, Hexal, Novartis, Pharmacosmos, Respicardia, Roche, Servier, Sorin and Vifor. SvH reports research support from Amgen, Boehringer Ingelheim, IMI and the German Centre for Cardiovascular Research (DZHK).
